# Ubiquitin-Specific Protease 5 Is Required for the Efficient Repair of DNA Double-Strand Breaks

**DOI:** 10.1371/journal.pone.0084899

**Published:** 2014-01-14

**Authors:** Satoshi Nakajima, Li Lan, Leizhen Wei, Ching-Lung Hsieh, Vesna Rapić-Otrin, Akira Yasui, Arthur S. Levine

**Affiliations:** 1 Department of Microbiology and Molecular Genetics and University of Pittsburgh Cancer Institute, University of Pittsburgh School of Medicine, Pittsburgh, Pennsylvania, United States of America; 2 Division of the Dynamic Proteome, Institute of Development, Aging, and Cancer, Tohoku University, Sendai, Japan; Tulane University Health Sciences Center, United States of America

## Abstract

During the DNA damage response (DDR), ubiquitination plays an important role in the recruitment and regulation of repair proteins. However, little is known about elimination of the ubiquitination signal after repair is completed. Here we show that the ubiquitin-specific protease 5 (USP5), a deubiquitinating enzyme, is involved in the elimination of the ubiquitin signal from damaged sites and is required for efficient DNA double-strand break (DSB) repair. Depletion of USP5 sensitizes cells to DNA damaging agents, produces DSBs, causes delayed disappearance of γH2AX foci after Bleocin treatment, and influences DSB repair efficiency in the homologous recombination pathway but not in the non-homologous end joining pathway. USP5 co-localizes to DSBs induced by laser micro-irradiation in a RAD18-dependent manner. Importantly, polyubiquitin chains at sites of DNA damage remained for longer periods in USP5-depleted cells. Our results show that disassembly of polyubiquitin chains by USP5 at sites of damage is important for efficient DSB repair.

## Introduction

DNA double-strand breaks (DSBs) are highly cytotoxic lesions generated by ionizing radiation and various DNA damaging agents. If they are not repaired or are repaired incorrectly, DSBs cause cell death or chromosomal instability which eventually leads to tumorigenesis or premature aging [1], [2]. The major repair pathways of DSBs in eukaryotic cells are the nonhomologous end-joining (NHEJ) and the homologous recombination (HR) pathways. NHEJ is active throughout the cell cycle, while HR is normally restricted to S and G2 cells because HR utilizes identical sister chromatids for repair. Like repair pathways active with other types of DNA damage, DSB repair requires the regulation of proteins by post-translational modification [3]. Although many proteins are post-translationally modified in DSB repair, a key modification is that of core histones surrounding DNA damage sites [4]. Although multiple histone modifications (phosphorylation, ubiquitination, sumoylation, methylation, acetylation) contribute to efficient DSB repair [5], phosphorylation and ubiquitination of core histones and sumoylation play the most important roles in this repair [6]-[8]. Following DNA damage, ATM phosphorylates the histone variant H2AX surrounding the damage site [9]. Then, RNF8-UBC13 mediates the ubiquitination of proteins at the damage site. RNF168-UBC13 recognizes the RNF8-mediated ubiquitinated protein and ubiquitinates H2A-type histones. RNF8-UBC13 extends the ubiquitination signal and allows the formation of K63-linked polyubiquitin chains [10]. The polyubiquitin chain is required for recruitment of downstream checkpoint and repair factors, including RAP80/BRCA1, 53BP1, and RAD18 [11]. In the process of conjugation of ubiquitin to a target protein, ubiquitin E3 ligase is the most important player because it confers substrate specificity and in most cases, determines the extent of ubiquitination and the type of linkage.

Like other modifications, ubiquitination of a target protein is a reversible reaction [12]. Ubiquitin modifications can be reversed by the action of deubiquitinating enzymes (DUBs). DUBs are ubiquitin-specific proteases that can remove the ubiquitin moiety from a target protein by editing or disassembling the polyubiquitin chain. DUBs are involved at several stages of the ubiquitination process. By removing the polyubiquitin signal from target proteins, DUBs can protect K48-linked polyubiquitin-conjugated proteins from degradation by the proteasome [13], [14]. DUBs also turn off a signal induced by the monoubiquitination of target proteins [15], [16], and they are involved in disassembling ubiquitin chains to regenerate free ubiquitin for re-use by the conjugation system. Thus, the ubiquitination process is regulated by a cooperative action of ubiquitin E3 ligases and DUBs.

Although the ubiquitination induced by DNA DSBs is well known, little is known about how the ubiquitination signal is eliminated from the damage sites. To gain further insight into the deubiquitination process and its effects on DSB repair, we investigated one of the DUBs. Here we show that USP5 (also known as isopeptidase T; ISOT) is a novel factor functioning in the repair of DSBs via HR. We also provide evidence suggesting that the disassembly of free polyubiquitin chains at damage sites, mediated by USP5, is necessary for efficient DSB repair.

## Materials and Methods

### Cells and culture conditions

Flp-In T-REx 293 cells (Invitrogen) were used for expression of FLAG-His-tagged USP5 or EGFP-tagged RAD18. HeLa cells were used for survival, the γH2AX foci formation assay, and the RAD51 foci formation assay with or without siUSP5 treatment. RAD18-deficient human cells were derived from HCT116 as previously described [17], [18]. U2OS SceI cells were previously described [19]. These cells were maintained in DMEM containing 10% of FBS with or without 1 mM of tetracycline for induction of expression.

### Plasmids

The human USP5 open reading frame was amplified by PCR from a cDNA (Open Biosystems, MHS1011-60809) using PCR primers with an Xho I site at the 5′ terminus (USP5 5′ Xho) and a Not I site at the 3′ terminus (USP5 3′ Not) and cloned into pBluescriptII. The identity of the cloned gene was confirmed by sequencing. There are two known alternatively spliced forms of USP5 which differ by an insertion of 23 amino acids. The substrate specificity of the isoforms appears to be identical *in vitro* [20]. We used transcription variant 2 (short isoform). Enhanced green fluorescent protein (EGFP)-tagged USP5 was generated by an in-frame ligation of a USP5 fragment (4–2505 nt) encoding the entire USP5 sequence, except for the start and termination codons, into either pEGFP-C1 or N1 (Clontech). To generate USP5 deletion mutants, USP5 was amplified by PCR with the primers. All of the constructs were confirmed by sequencing. EGFP-tagged ubiquitin was generated as above. The pDsRed-Monomer-RAD18 was previously described [17]. pCherry-TA was derived from pCherry-TA-ER [19] but the ER domain was removed.

### Antibodies and siRNA

Anti-FLAG (M2; Sigma), anti-GFP (clones 7.1 and 13.1; Roche), anti-USP5 (BC005139; Proteintech Group, Inc.), anti-RAD18 (A301-304A; BETHYL), anti-ubiquitin FK1, FK2 (Cosmo Bio), anti-Rad51 (Cell Signaling), and anti-phospho histone H2AX (Ser139) (Millipore) were used. A synthetic siRNA duplex (D-006095-02, -03 and -04) for USP5 was purchased from Dharmacon. The synthetic siRNA duplex (AM16708) for RAD18 was purchased from Ambion. Both siRNA duplexes were used previously [21], [22]. Ambion *In Vivo* Negative Control #1 siRNA was used as a control siRNA. When we treated cells with the transfection reagent but without adding siRNA for a control, we indicated this as “-siRNA.” When we transfected the negative control siRNA, we indicated this as “+siNC.”

### Survival assay

Cells were plated at 2×10^5^ cells per 60-mm Petri dish and cultured overnight. Cells were transfected with or without 2 nM siRNA for USP5 by using DharmaFECT (Thermo). After 3 days, these cells were subjected to the survival assay. Cells were plated at 200 cells per 60-mm Petri dish and treated with methyl methanesulfonate (MMS) (Sigma), Bleocin (Calbiochem), or hydroxyurea (HU) (Sigma). Cells were cultured after treatment for 12–14 days. Colonies were fixed and stained with 0.3% crystal violet in methanol, and the number of colonies was counted.

### Laser micro-irradiation

Laser micro-irradiation was performed as previously described [19], [23]. Briefly, cells were plated at 1×10^5^ cells per 35-mm glass bottom dish (MatTek) and cultured at least overnight. For laser micro-irradiation, cells were treated with or without 100 mM 8-MOP for 10 min prior to irradiation with laser light of 405 nm, which is not utilized by 8-MOP for cross-link formation, but has a sensitizing effect on DNA [24]. The irradiation dose was 5 mW (100%) for 10 or 100 ms at a single point irradiation. The same dose was applied for 10 or 100 frames at a single line irradiation. After irradiation, cells were incubated in medium for various periods of time as indicated and then fixed and stained. For analysis of deletion mutants of USP5, at least 33 cells were irradiated and analyzed. After irradiation, we measured the intensity of EGFP fluorescence at the irradiated area and compared the intensity to that of the unirradiated area. We defined it as a foci positive cell if the intensity of the irradiated area is increased more than 1.2 fold compared to that of the unirradiated area. The ATM inhibitor (KU-55933; Selleckchem) was added for 2 hr prior to irradiation, and the final concentration was 10 µM. The PARP inhibitor (Olaparib; Sigma) was added for 30 min prior to irradiation, and the final concentration was 10 µM.

### Enzymatic production of DSBs at the restricted area of cell nuclei

U2OS SceI cells were plated at 2×10^5^ cells per 35-mm glass-bottom dish and cultured overnight. Cells were transfected with pCMV-NLS-I-SceI and with or without USP5-EGFP and incubated for 40 hr. Cells were fixed and stained by anti-γH2AX antibody with or without anti-USP5 antibody. In the case of endogenous USP5, three independent images were taken, and cell nuclei which contained USP5 foci co-localized with γH2AX foci were counted. At least 150 cells were counted in each sample.

### γH2AX foci formation assay

Cells were plated at 2×10^5^ cells per 35-mm glass-bottom dish and cultured overnight. Cells were transfected with or without siUSP5 and incubated for 40 hr. Cells were treated with 5 mg/ml Bleocin for 1 hr and incubated for the time indicated. The number of γH2AX foci positive cell nuclei was counted after staining with anti-phospho histone H2AX. We define a cell nucleus which contains more than five γH2AX foci as a foci positive cell.

### NHEJ and HR assays

NHEJ and HR assays were previously described [19]. Briefly, to express I-SceI, pCMV-NLS-I-SceI was introduced by transfection, using Lipofectamine 2000 reagent, into 1.5–3×10^5^ H1299 dA3-1#1 cells (for NHEJ) [25] or 3×10^5^ HeLa pDR-GFP cells (for HR) [26] pretransfected with siRNA for 48 hr using Lipofectamine RNAiMAX (Invitrogen). siBRM for the NHEJ assay or siBRCA1 for the HR assay was used as a positive control [25]. EGFP-positive cells were counted with Cellquest software. For FACS analysis, cells were harvested by trypsinization, washed with PBS, stained, and applied on the FACS caliber apparatus (Becton Dickinson).

### RAD51 foci formation assay

HeLa cells were plated at 1×10^5^ cells per 35-mm glass-bottom dish and cultured overnight. Cells were transfected with or without siUSP5-03 or -04 and incubated for 48 hr. Cells were treated with X-ray from a ^137^Cs source with total doses of 5 Gy (fluxes of 0.68 Gy/min) and incubated for the time indicated. RAD51 foci positive cells were counted in 50 cells in each of three different areas. We define a cell nucleus which contains more than three RAD51 foci as a foci positive cell.

### Primers

To generate USP5 expression constructs, USP5 was amplified by PCR with the following primers:

USP5 5′ Xho: TTT TCT CGA GGC GGA GCT GAG TGA GGA GGC GCT G; USP5 3′ Not: TTT TGC GGC CGC AGC TGG CCA CTC TCT GGT AGA AGT; USP5 5′ Xho 571: TTT TCT CGA GCG AAT CCC TCC CTG TGG CTG GAA G; USP5 5′ Xho 817: TTT TCT CGA GGC TGA GCA CCT GTC CCA CTT CGG C; USP5 5′ Xho 2089: TTT TCT CGA GGC CGA CCC CCC TCC TGA GGA CTG T; USP5 3′ Not 594: TTT TGC GGC CGC ACT TCC AGC CAC AGG GAG GGA TTC G; USP5 3′ Not 840: TTT TGC GGC CGC AGC CGA AGT GGG ACA GGT GCT CAG C; USP5 3′ Not 2112: TTT TGC GGC CGC AAC AGT CCT CAG GAG GGG GGT CGG C.

### Immunofluorescence

After irradiation, cells were incubated in medium for the indicated time and then washed twice with PBS and fixed with methanol-acetone (1∶1) for 10 min at −20°C. The fixed cells were dried, then rinsed once with PBS-T (0.05% Tween 20 in PBS), and incubated in 5% BSA in PBS-T for 30 min at room temperature. Cells were then incubated with antibody for more than 1 hr. Cells were washed 3 times with PBS-T and incubated with a second antibody conjugated with Alexa Fluor (Molecular Probes) for more than 30 min. Cells were washed 3 times with PBS-T and then mounted in drops of VECTASHIELD Mounting Medium with DAPI (Vector Laboratories).

## Results

### USP5 responds to DNA DSBs

**Figure 1:**
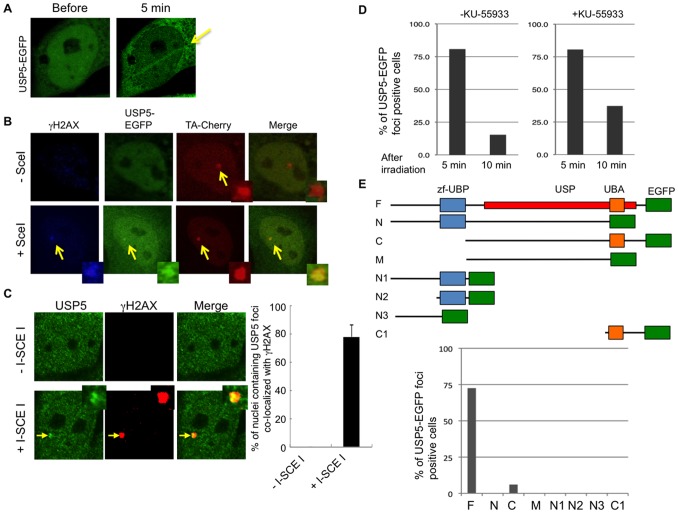
USP5 co-localizes with DNA DSBs. A: EGFP-tagged USP5 was expressed in HeLa cells, and cells were irradiated with the laser at a dose of 100 frames with 100 µM of 8-MOP. 8-MOP sensitizes laser light to produce DNA base damage and strand breaks. **B**: EGFP-tagged USP5 co-localizes with DNA DSBs produced by a restriction enzyme. Plasmid DNAs for expression of USP5-EGFP and Cherry-TA were introduced in U2OS SceI cells with or without NLS-SceI expression plasmid DNA by Lipofect amine 2000. After overnight incubation, cells were fixed and stained by anti-γH2AX antibody. **C**: Endogenous USP5 co-localizes with DNA DSBs produced by a restriction enzyme. Plasmid DNA for expression of NLS-SceI was introduced in U2OS SceI cells by Lipofect amine 2000. After overnight incubation, cells were fixed and stained by anti-USP5 antibody and anti-γH2AX antibody. At least 150 cells were counted in each sample. **D**: Damage response of USP5-EGFP after laser micro-irradiation with or without ATM inhibitor. Cells were irradiated with the laser at a dose of 100 frames with 100 µM of 8-MOP and with or without 10 µM of ATM inhibitor. The results are averages obtained from two independent experiments, and more than 52 cells were irradiated and analyzed for the damage response. **E**: Domain analysis of USP5 with regard to the damage response. Schematic presentation of domains in USP5 and the GFP-tagged mutant constructs. zf-UBP, Zinc-finger in ubiquitin-hydrolases and other proteins; UBA, Ubiquitin Associated domain. EGFP-tagged USP5 full length or deletion mutant was expressed in HeLa cells, and cells were irradiated with the laser at a dose of 100 frames with 100 µM of 8-MOP. The results are averages obtained from at least two independent experiments and more than 33 cells were irradiated and analyzed.

USP5 is one of the DUBs and it disassembles unanchored polyubiquitin chains by a sequential exo mechanism [27]. To investigate whether USP5 is involved in the DDR, we analyzed the response of EGFP-tagged USP5 to DNA DSBs. Here, we found that EGFP-tagged USP5 co-localized to laser micro-irradiation sites (Figure 1A). The C-terminus fusion of USP5 (USP5-EGFP) co-localized to sites of DNA damage induced by laser micro-irradiation, while the N-terminus fusion of USP5 (EGFP-USP5) did not co-localize. The radiation dose is relatively high enough to produce DNA DSBs, suggesting that USP5 may co-localize with DSBs. To confirm that USP5 co-localizes to DNA DSBs, we used another cell line, U2OS SceI (Figure S1 in File S1) [19]. U2OS SceI cells harbor a stable 200 copy transgene array of a plasmid containing the restriction site for I-SceI adjacent to a 96 repeat array of tetracycline-response-elements (TREs). If we express I-SceI endonuclease, DSBs are induced enzymatically at the restricted area of cell nuclei which co-localizes a Cherry-TA focus. As shown in Figure 1B, the USP5-EGFP focus was clearly detected if I-SceI endonuclease was introduced simultaneously, although we could not detect USP5 foci that co-localized to γH2AX (a marker of DSBs) foci after Bleocin treatment. Next, to determine whether endogenous USP5 also responds to DSBs, we investigated the damage response of endogenous USP5 by using anti-USP5 antibody (Figure 1C). Without expression of I-SceI endonuclease, we could not detect USP5 foci (Figure 1C, upper panel). However, with expression of I-SceI endonuclease, we clearly detected USP5 foci that co-localized to γH2AX foci (Figure 1C, lower panel). About 80% of γH2AX foci co-localized to USP5 foci (Figure 1C, left). These data indicate that USP5 responds to DSBs.

We counted foci positive cells at 5 min and 10 min after irradiation. About 80% of the cells were foci positive at 5 min after irradiation (Figure 1C, left panel). Foci positive cells gradually decreased to about 15% at 10 min after irradiation. To investigate whether the damage response of USP5 depends on ATM activity, we examined the damage response of USP5 with the ATM inhibitor, KU-55933. Although the frequency of γH2AX foci positive cells after Bleocin treatment was clearly decreased by treatment with KU-55933 (Figure S2 in File S1), the frequency of USP5-EGFP foci positive cells at 5 min after laser irradiation with treatment of KU-55933 was almost the same as that of foci positive cells without treatment with KU-55933 (Figure 1C, right panel). The frequency of USP5-EGFP foci positive cells slightly increased at 10 min after irradiation in the presence of KU-55933. The delayed disappearance may be caused by the inhibition of DSB repair in the presence of the ATM inhibitor. The data indicate that the damage response of USP5 does not depend on ATM activity. Since many repair proteins co-localize to DNA damage in a PARP-dependent manner [28], we also examined the influence of the PARP inhibitor, Olaparib (AZD2281, KU0059436). However, the damage response of USP5 was not affected by the PARP inhibitor (Figure S3 in File S1).

To determine which domain of USP5 is required for co-localization of USP5 with DSBs, we examined co-localization of deletion mutants with DSBs. Although the C mutant co-localized with DSBs slightly, the co-localizations are weak and the frequency is low compared to that of full length USP5. Only the full length USP5 co-localizes with DSBs stably and strongly (Figure 1D). We conclude that full length USP5 is required for efficient co-localization with DSBs induced by laser micro-irradiation.

We also examined the damage response of USP5 to different types of damage, UV-induced DNA damage [29], [30] or single strand breaks induced by UV damage endonuclease and UV irradiation [31]. We did not detect co-localization of USP5 to either type of DNA damage.

### USP5 is necessary for cell survival after DNA damage

**Figure 2:**
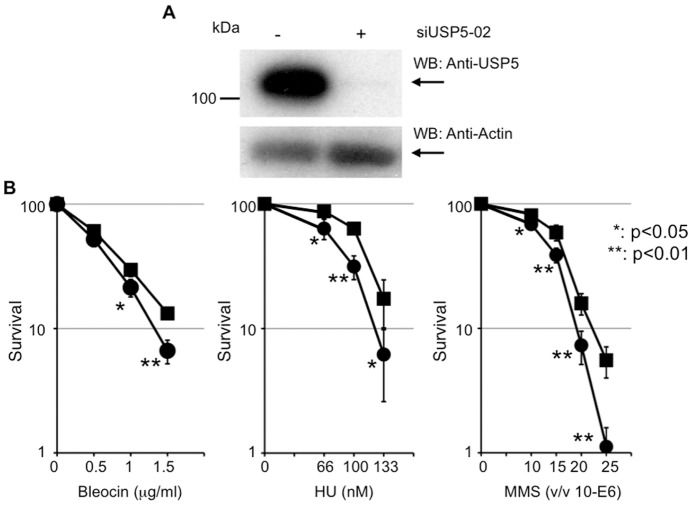
USP5 is necessary for cell survival after DNA damage. A: Knockdown of USP5 expression by siUSP5 treatment. **B**: Colony forming assay after treatment with Bleocin, hydroxyurea, or methyl-methanesulfonate, with or without siUSP5 treatment. Filled square indicates without siUSP5 treatment and filled circle indicates with siUSP5 treatment; error bars, ± SED. The *P*-value was calculated using Student's *t*-test.

To investigate whether USP5 is required for DNA repair, we examined cell survival in USP5-depleted cells after treatment with various DNA damaging reagents. siUSP5 suppressed USP5 expression to about 10% of control (Figure 2A), and this treatment sensitized cells to Bleocin, hydroxyurea (HU), and methyl-methanesulfonate (MMS) significantly (Figure 2B). Thus, USP5 is necessary for cell survival after DNA damage. Although depletion of USP5 sensitized cells to DNA damaging agents, the effect is relatively mild. Since we considered that this mild effect was caused by insufficient and/or temporary suppression of USP5 by siRNA, we tried to establish a constitutive cell line using shRNA. Although we identified several clones whose expression of USP5 was suppressed, the effect of suppression was reduced during culture and the expression level of USP5 reached a normal level. Constitutive suppression of USP5 may not be achieved in the cell because of collapse of the ubiquitin system.

### USP5 knockdown causes the delayed disappearance of γH2AX foci

**Figure 3:**
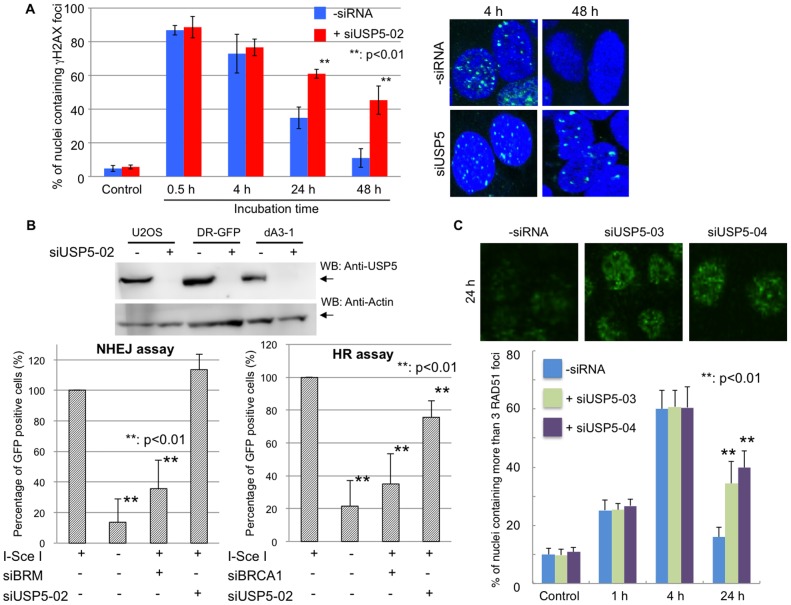
USP5 is required for double strand break repair via the homologous recombination pathway. A: USP5 knockdown affects the disappearance of γH2AX foci. The number of nuclei which contain more than 5 γH2AX foci was counted and summarized in the graph. Blue bar indicates without siUSP5 treatment and red bar indicates with siUSP5 treatment; error bars, ± SED. The *P*-value was calculated using Student's *t*-test. Representative figures for γH2AX foci after Bleocin treatment in the presence and absence of siUSP5 are shown at the left. **B**: NHEJ and HR frequencies in cells depleted of USP5. Results of western blot analysis after siRNA treatment are shown at the left. Assay for NHEJ of chromosomal DSBs in H1299 cells or assay for HR frequency of chromosomal DNA containing a recombination substrate DR-GFP in HeLa cells. The GFP-positive cell fraction in cells depleted of USP5 was determined and compared with that in cells treated with siCont or siBRM for the NHEJ assay or siBRCA1 for the HR assay for determination of frequencies; error bars, ± SED. The *P*-value was calculated using Student's *t*-test. **C**: USP5 knockdown affects the disappearance of RAD51 foci. Representative figures for RAD51 foci after 5 Gy X-ray irradiation in the presence and absence of siUSP5 are shown at the top. The number of nuclei which contain more than three RAD51 foci was counted and summarized in the graph.

USP5-depleted cells showed sensitivity to the DNA damage reagents, which are producing DSBs directly or indirectly, and USP5 co-localizes to DSBs after laser micro-irradiation, suggesting that USP5 may play a role in DSB repair. To investigate whether USP5 is required for DSB repair, we monitored the appearance and disappearance of γH2AX foci after Bleocin treatment with or without siUSP5 treatment (Figure 3A). Without Bleocin treatment or with the treatment at 0.5 hr or 4 hr post-incubation, there were no significant differences with or without siUSP5 treatment. This indicates that USP5 is not required for the maintenance of the steady state level of γH2AX foci or the production of γH2AX foci after DNA damage. In control cells, the number of γH2AX foci positive cells decreased at 24 hr and 48 hr post incubation, showing that DSBs are repaired in these cells. However, USP5-depleted cells delayed the disappearance of γH2AX foci significantly at 24 hr and 48 hr post-incubation. In these cells, DSBs are not repaired efficiently. Thus, USP5 is required for efficient DSB repair.

### Depletion of USP5 influences HR but not NHEJ

DNA DSBs are repaired by two different pathways in human cells, HR and NHEJ. To investigate which pathway USP5 is involved in, we analyzed HR and NHEJ frequencies by using reporter assays (Figure 3B) [25], [26]. Although it is not comparable to the effects caused by BRCA1 depletion (<40%), depletion of USP5 expression reduced the percentage of GFP-positive cells to less than 80% as compared with control cells in the HR assay (Figure 3B, left panel). However, depletion of USP5 expression did not reduce the percentage of GFP-positive cells at all in the NHEJ assay (Figure 3B, right panel). These data indicate that USP5 is involved in the repair of DSBs via the HR pathway but not the NHEJ pathway. It is known that the repair frequency of HR depends on cell cycle stages. siUSP5 treatment may affect normal cell cycle progression and cause the suppression of HR. To exclude this possibility, we analyzed cell cycle progression after siUSP5 treatment by FACS, finding that siUSP5 treatment does not affect the distribution of cells in different cell cycle stages compared to control (Figure S4 in File S1). To exclude another possibility, that the defect of HR is caused by siRNA treatment itself or off-target effects from use of only one single USP5 siRNA (siUSP5-02), we confirmed the effect of the negative control siRNA (siNC) or two independent siRNAs targeting USP5 (siUSP5-03, -04) on cell survival or the HR assay (Figure S5 in File S1). Although siUSP5-04 treatment showed a relatively severe phenotype, three independent siRNA treatments caused an almost similar phenotype. We also checked the expression level of RAD51 because RAD51 is easily affected by the off-target effects [32]. There are no significant differences in the expression level of RAD51 in siRNA-treated cells. These data suggest that the defect of HR is not caused by the off-target effects of siRNA treatment.

### Depletion of USP5 influences the disappearance of RAD51 foci after X-ray irradiation

Since depletion of USP5 influences HR, we investigated the disappearance of RAD51 foci after X-ray irradiation with or without siUSP5 treatment (Figure 3C). Depletion of USP5 does not affect the number of RAD51 foci in the cells analyzed 1 hr or 4 hr after X-ray treatment, suggesting that USP5 is not required for the production of RAD51 foci after DNA damage. The number of RAD51 foci positive cells without siRNA treatment decreased at 24 hr post-incubation compared to 4 hr post-incubation, while USP5-depleted cells delayed the disappearance of RAD51 foci significantly at 24 hr post-incubation. These data support the conclusion that USP5 plays an important role in HR.

### USP5 is required for rapid dissociation of ubiquitin at sites of DSBs

**Figure 4:**
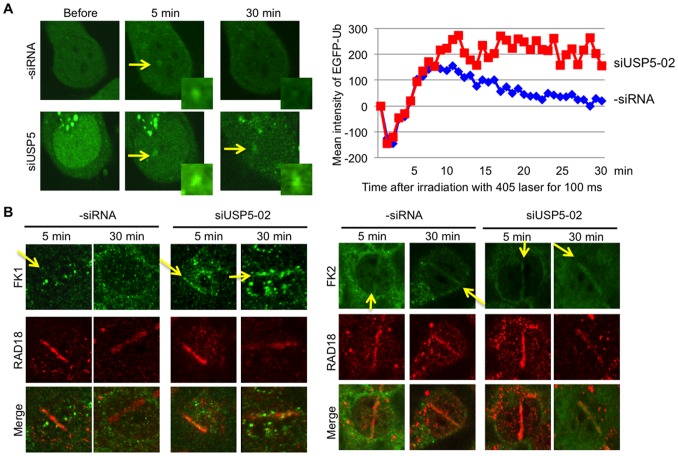
USP5 is required for disassembly of polyubiquitin chains at sites of DNA damage. A: EGFP-tagged ubiquitin was expressed in HeLa cells with or without siUSP5 treatment. Cells were irradiated with the laser light for 100 ms. The intensity of EGFP-Ub at the irradiated site was analyzed and summarized in the graph. The results are averages obtained from three independent experiments (n = 5). **B**: Cells were irradiated with the laser for 10 frames with 100 µM of 8-MOP and then fixed and stained by anti-ubiquitin antibody (FK1; left panel, FK2; right panel) and anti-RAD18 antibody. Anti-RAD18 antibody is used for showing irradiated sites.

Since many proteins are monoubiquitinated or polyubiquitinated at sites of DSBs, we speculated that the role of USP5 at sites of damage is to eliminate polyubiquitin chains induced by DSBs. To investigate whether depletion of USP5 affects the kinetics of ubiquitin at sites of damage, we expressed EGFP-tagged ubiquitin (EGFP-Ub) in cells and irradiated the cells with the laser. In control cells, EGFP-Ub co-localized with damage immediately, reaching a peak around 10 min after irradiation and then dissociating from damage sites gradually (Figure 4A, blue line). In USP5-depleted cells, EGFP-Ub co-localized with damage immediately as in control cells but did not dissociate from damage sites even 30 min after irradiation (Figure 4A, red line). These data indicate that depletion of USP5 delays dissociation of ubiquitin from damage sites but does not affect the recruitment of ubiquitin to damage sites. However, it is impossible to distinguish between monoubiquitination and polyubiquitination by this method. To determine whether the increased signal is caused by accumulation of a monoubiquitinated protein or a polyubiquitin chain, we used a ubiquitin antibody, FK1, which recognizes the polyubiquitin chain but not monoubiquitinated protein or free ubiquitin. Although the signal of polyubiquitination is very faint without siUSP5 treatment, we detected the signal at damage sites at 5 min after irradiation (Figure 4B, left panel). At 30 min after irradiation, the signal was not detected at damage sites. In contrast, the signal of polyubiquitination was detected even at 30 min after irradiation with siUSP5 treatment. These data are well correlated with the data obtained by EGFP-Ub. We also used another ubiquitin antibody, FK2, which recognizes both polyubiquitin chains and monoubiquitinated proteins. There is no significant difference among signals detected by FK2 (Figure 4B, right panel). Since the preferred substrate for USP5 is an unanchored polyubiquitin chain, in other words, the free polyubiquitin chain [27], [33], [34], it is plausible that the increased signal obtained by FK1 antibody in USP5-depleted cells is caused by accumulation of free polyubiquitin chains at sites of damage. Thus, USP5 is apparently required for the rapid elimination of free polyubiquitin chains from damage sites.

### USP5 interacts with RAD18

**Figure 5:**
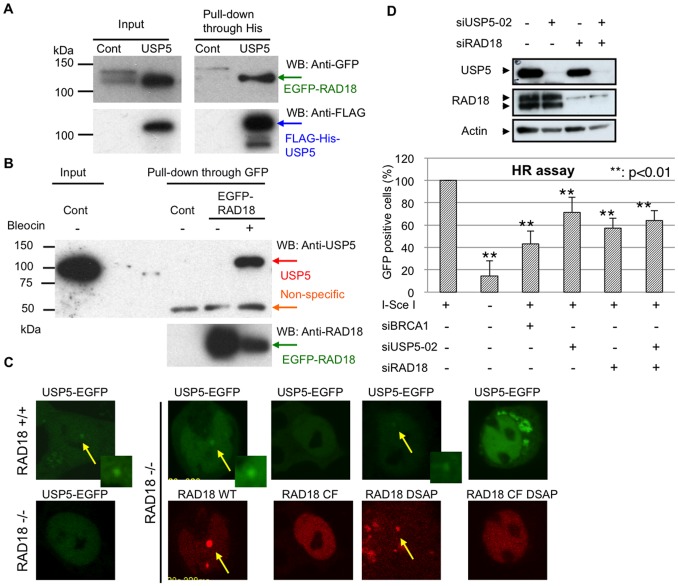
USP5 interacts with RAD18 and USP5 depends on RAD18 in DSB repair. A: Interaction between expressed proteins. We transiently expressed EGFP-tagged RAD18 in cells expressing FLAG-His-tagged USP5 and pulled down by the His-tag and pull downs were detected with anti-GFP antibody. **B**: Interaction between EGFP-tagged RAD18 and endogenous USP5. Cells were treated with or without Bleocin for 2 hr and then cells were extracted. Extracts were pulled down with anti-GFP antibody and detected by anti-USP5 antibody. **C**: Damage response of USP5-EGFP after laser micro-irradiation in RAD18-proficient or -deficient cells. EGFP-tagged USP5 and DsRed-tagged RAD18 WT or each mutant were co-expressed in RAD18-deficient cells, and the damage response after laser micro-irradiation was analyzed. Cells were irradiated with the laser light for 100 ms. **D**: HR frequencies in cells depleted of USP5 and/or RAD18. Results of western blot analysis after siRNA treatment are shown on the top. The GFP-positive cell fraction in cells depleted of USP5 and/or RAD18 was determined and compared with that in cells treated with siCont or siBRCA1 for determination of frequencies; error bars, ± SED. The *P*-value was calculated using Student's *t*-test.

Some DUBs appear to function with high specificity toward one or several substrates, but there are only a few examples of this specificity. The functional specialization of DUBs is associated with their residence in specific protein complexes [35]. DUBs and E3 are often found in a complex together [36], [37]. Although we showed that full length USP5 co-localized with DNA damage induced by laser micro-irradiation, there is no domain that is known to have the specific ability to respond to DNA damage in USP5, like a BRCT domain or zinc-finger domain [3]. From the above facts and results, we speculated that the E3 ligase complex or another protein involved in DSB repair might recruit USP5 to sites of damage. We investigated the interaction between USP5 and several E2–E3 ligase complexes that are known to be involved in DSB repair by pull-down experiments between USP5 and RNF168, UBC13, BRCA1, and RAD18. Among these E2–E3 ligase complexes, we found that USP5 interacts with RAD18. RAD18 is highly conserved from yeast to human and plays a major role in post-replication repair (PRR). RAD18 interacts with HHR6A, B (E2) and Pol eta (translesion polymerase) and regulates the polymerase switch via monoubiquitination of PCNA [38], [39]. In vertebrates, RAD18 has another role besides PRR [40]. RAD18 is also involved in DSB repair via the HR pathway and recruits RAD51C to DSBs [41]. We transiently expressed EGFP-tagged RAD18 in cells expressing FLAG-His-tagged USP5 and pulled it down by the His-tag. EGFP-tagged RAD18 was significantly pulled down in the cells expressing FLAG-His-tagged USP5 (Figure 5A). Next, to confirm the interaction, we checked the binding between EGFP-tagged RAD18 and endogenous USP5. We detected a clear interaction when EGFP-RAD18 was pulled down, and the pull downs were detected by anti-USP5 antibody only in the presence of Bleocin treatment (Figure 5B). This data indicates that USP5 interacts with RAD18 in the presence of DNA damage.

### RAD18 recruits USP5 to DSBs

To investigate whether RAD18 is required for co-localization of USP5 with DSBs, we analyzed the damage response of USP5 after laser micro-irradiation in RAD18-proficient or -deficient cells. Although co-localization of USP5 is observed clearly in RAD18-proficient cells, co-localization of USP5 is significantly reduced in RAD18-deficient cells (Figure 5C, left panel). This data indicates that efficient co-localization of USP5 to DSBs depends on RAD18. We have previously shown that RAD18 responds to UV-induced lesions and DNA strand breaks independent of DNA replication, and this ability depends on the zinc-finger domain of RAD18 [17]. Other groups also have shown that the zinc-finger domain of RAD18 binds to polyubiquitin chains [42] and RAD18 recruits to DSBs in a zinc-finger domain dependent manner [41]. The middle part of RAD18 contains another damage responsive domain, the SAP domain, in addition to the zinc-finger domain. The SAP domain is responsible for recruitment to stalled replication forks [17], [43]. To investigate further, we transiently co-expressed EGFP-tagged USP5 and DsRed-tagged RAD18 WT or mutants in RAD18-deficient cells and examined co-localization of USP5 after laser micro-irradiation (Figure 5C, right panel). Expression of the DsRed vector alone did not result in co-localization of USP5 with DSBs in RAD18-deficient cells, while expression of DsRed fused to RAD18 WT clearly resulted in co-localization of USP5 with DSBs (Figure 5C, right panel). The zinc-finger mutant of RAD18 (RAD18 CF) does not co-localize with DSBs, and expression of the zinc-finger mutant does not result in co-localization of USP5 with DSBs. On the other hand, the SAP domain deletion mutant (RAD18 ΔSAP) co-localizes with DSBs, and expression of the SAP domain deletion mutant results in co-localization of USP5 with DSBs. As expected, the double mutant (RAD18 CF ΔSAP) does not co-localize with DSBs, and expression of the double mutant does not result in co-localization of USP5. These data indicate that co-localization of USP5 with DSBs depends on RAD18 and its zinc-finger domain.

### USP5 plays a role in the same pathway as RAD18 to repair DSBs

Since efficient co-localization of USP5 with DSBs depends on the damage response of RAD18, we speculated that USP5 plays a role in the same pathway as RAD18 to repair DSBs. To investigate whether USP5 is required in the same pathway as RAD18, we analyzed HR frequencies depleted of USP5 and/or RAD18 (Figure 5D). Depletion of RAD18 expression reduced the significant percentage of GFP-positive cells to less than 60% of control cells in the HR assay. Although there may be a slight difference between the effects caused by RAD18 depletion (<60%) and USP5 depletion (<80%), the difference is not significant. Depletion of both expressions did not further reduce the percentage of GFP-positive cells, indicating that there is no additive effect of depletion by siUSP5 and siRAD18 on HR frequency. Thus, the data suggest that USP5 belongs to the same epistasis group as RAD18 in DSB repair.

## Discussion

Ubiquitination of proteins surrounding a DSB is one of the key steps in DSB repair and many proteins are mono- or polyubiquitinated near the sites of DSBs. While it is well known that several E3 ligases are involved in the ubiquitination of proteins associated with DSBs, little is known about the mechanism that removes the mono- or polyubiquitin from proteins at damage sites. We found that USP5 is a novel player involved in efficient HR repair. USP5 is one of a number of DUBs which have been studied well; the activity of USP5 concerning substrate specificity and kinetics is well understood [27], [33], [34]. However, the role of USP5 in DNA repair is not known. We found that depletion of USP5 results in increased sensitivity to DNA damaging agents (Figure 2B) and the delayed disappearance of γH2AX foci (Figure 3A), and this defect is associated with an impairment of HR (Figure 3B, C). These data suggest that USP5 plays a role in DSB repair via the HR pathway.

There is accumulating evidence that ubiquitination-mediated protein degradation at sites of damage, and recruitment of proteasomes to damage sites, are important for efficient DSB repair. A recent paper has shown that the SUMO-targeted ubiquitin E3 ligase, RNF4, is required for turnover of MDC1 and RPA1, and promotes DSB repair [44]. Another paper has shown that JMJD2A is a novel substrate of RNF8 and RNF168 for ubiquitination after DNA damage, and recruitment of 53BP1 to sites of DNA damage depends on degradation of JMJD2A [45]. These results indicate that degradation of proteins induced by ubiquitination at sites of damage is important for DSB repair. Moreover, several papers have shown that some components of the 26S proteasome are recruited to sites of damage and proteasome activity is required for efficient DSB repair [44], [46]. As shown in Figure 3, we found that USP5 is recruited to DSBs immediately after they are formed, and the recruitment depends on RAD18 (Figure 5). Moreover, we found that USP5 is required for rapid elimination of the polyubiquitin chain at sites of damage (Figure 4). These data suggest that USP5 also plays a role in regulating protein degradation at sites of damage by disassembling free polyubiquitin chains.

Depletion of USP5 only affected the frequency of HR repair but not NHEJ in DSB repair (Figure 3B). The mechanism of HR is more complicated than that of NHEJ. HR utilizes an identical sister chromatid as a template for accurate repair, while NHEJ connects broken ends directly. It is known that many repair proteins involved in HR form ionized radiation-induced foci (IRIF), indicating that a large number of molecules accumulate at one DSB [47]. In contrast, repair proteins involved only in NHEJ, such as DNA-PKcs, KU70/80, and XRCC4/LIG4, do not form IRIF, and detection of co-localization of these repair proteins with DSBs requires a large number of DSBs in a restricted area using laser micro-irradiation or I-SceI expression plus high copy number I-SceI recognition sites in the genome [19], [48], [49]. This fact indicates that a relatively low number of molecules are required for the repair of one DSB in the NHEJ pathway compared to HR. The turnover of protein at sites of damage may be more important in the HR pathway than the NHEJ pathway. Therefore, depletion of USP5 only affected the frequency of HR. However, DUBs other than USP5 might be required for the efficient repair of DSBs in the NHEJ pathway.

We showed that USP5 is required for rapid elimination of the polyubiquitin chains at sites of damage (Figure 4), and we considered the mechanisms by which polyubiquitin chains accumulate at sites of DNA damage in the absence of USP5. Although USP5 has high activity in disassembling free polyubiquitin chains from their free C-terminus end regardless of the linkage type of polyubiquitination, USP5 has almost no activity in cutting the bond between substrate protein and the C-terminus of ubiquitin [27], [34]. In order for USP5 to work, other DUB(s) must remove a polyubiquitin chain from the substrate protein. In humans, 3 DUBs associate with the 26S proteasome: POH1/RPN11, USP14, and UCH37 [35]. In order to recycle ubiquitin molecules, the polyubiquitin chain is removed before proteins are processed by proteasomes [50]. USP14 and UCH37 appear to antagonize degradation by removing the polyubiquitin chain from the substrate protein through cutting at the distal tip of the chain, whereas POH1/RPN11 appears to promote substrate degradation by cutting at the base of the chain to release the chain *en bloc* [35]. USP5 disassembles this free polyubiquitin chain efficiently. Recently, it has been shown that POH1/RPN11 is required for a DSB response [51]. Another possibility is that some DUBs remove polyubiquitin chains from substrate proteins without disassembly of the chains. It is known that several DUBs are involved in DSB repair. For example, USP11 and USP3 antagonize RNF8-mediated ubiquitination [52]–[54], and the USP1/UFA complex promotes DSB repair via HR [55]. The role of USP5 may then be to eliminate the free polyubiquitin chains from sites of DSBs.

Depletion of USP5 causes the accumulation of polyubiquitin chains at sites of damage, and this inhibits the efficient repair of DSBs (Figures 3, 4). This result suggests that polyubiquitin chains must be removed rapidly from damage sites. It is known that accumulation of polyubiquitin chains inhibits the proteasome activity by a competing mechanism in yeast and human cells [22], [56]. Accumulation of polyubiquitin chains at sites of damage may delay the turnover of proteins which should be degraded for proper progression of DSB repair. Another possibility is that free polyubiquitin chains recruit other repair proteins or retain repair proteins which have a ubiquitin-binding motif. This may inhibit progression of the repair process or the next round of repair. This may also explain why the depletion of USP5 has only a mild effect on survival and HR because USP5 controls protein degradation through the ubiquitin system but not the HR mechanism directly.

From the above results and facts, we propose the following model. After production of DSBs, many proteins surrounding damage sites, including core histones, are mono- or polyubiquitinated. This ubiquitination signal recruits many repair proteins. USP5 is also recruited to sites of DNA damage in a RAD18-dependent manner. During the repair process or after completion of repair, polyubiquitinated proteins are degraded by the proteasome. Alternatively, polyubiquitin chains are removed by some DUBs without disassembly. This causes accumulation of free polyubiquitin chains at sites of damage. The accumulation of polyubiquitin chains inhibits turnover of proteins at the sites of damage, and this may inhibit proper progression of the repair process or the next round of repair. Thus, USP5 is required for the efficient repair of DSBs by disassembling free polyubiquitin chains at sites of damage.

There are approximately 95 putative DUBs in human cells, whereas there are more than 600 putative E3 ligases, indicating the low substrate specificity of DUBs compared to E3 ligases [36], [57]. The functional specialization of DUBs often reflects their residence in specific protein complexes [35]. The action of USP5 is the disassembly of free polyubiquitin chains. This activity is general rather than the removal of ubiquitin from specific substrate proteins. However, by binding to RAD18, which recognizes polyubiquitin chains, USP5 is recruited to sites of damage and plays a role in DSB repair, disassembling polyubiquitin chains at the sites of damage. Our results suggest how the ubiquitination process is regulated by protein-protein interactions to confer specific roles to a relatively small number of DUBs.

## Supporting Information

File S1 **Figures S1–S5.** Figure S1. U2OS I-SceI system for inducing DSB in a single cell; Enzymatic production of DSB at a restricted area of the nucleus. An I-SceI site was inserted next to 96 repeats of TRE. The length of an I-SceI site is 18 base pairs and there is no I-SceI site in the human genome. We introduced this construct into a U2OS cell and selected a clone which has more than 200 copies of the construct in its genome at only one position. To show the position where this construct is inserted, Cherry-TA (Cherry is a red fluorescent protein) and TA (a trans-activator which binds to TRE) were used. When we express Cherry-TA, the spot which shows the position of the construct is detected as a red focus. With expression of I-SceI, a γH2AX focus colocalized with a Cherry focus is detected. With expression of I-SceI and a GFP-tagged repair protein, Ku80, a GFP-Ku80 focus colocalized with a Cherry focus is detected. Figure S2. Treatment with an ATM inhibitor affects the foci formation of γH2AX after bleocin treatment. A: Cells were treated with 5 µg/µl of bleocin for 1 hr with or without the ATM inhibitor, KU-55933, and then cells were washed twice and fresh medium was added with or without KU-55933. After incubation for 2 hr, cells were fixed and stained with anti-phospho histone H2AX. Representative data are shown. B: % of γH2AX foci positive cells was summarized in the graph. The number of cell nuclei which contain more than five γH2AX foci was counted as foci positive cells. In each condition, more than 300 cells were counted. Figure S3. Treatment with the PARP inhibitor, Olaparib, does not affect the damage response of USP5 after laser irradiation. Percent of USP5-EGFP foci positive cells at 5 min after irradiation was summarized in the graph. The result was obtained in two independent experiments and more than 50 cells were irradiated and analyzed. Figure S4. siUSP5 treatment does not affect cell cycle progression. Cells were treated with or without siUSP5. After 3 days, cells were fixed with 70% ethanol and stained by PI (Propidium iodide) and then analyzed with a C6 Flow Cytometer (Accuri Cytometers, Inc.). For a positive control, cells were treated with HU (1 mM) or nocodazole (100 µg/ml) for 24 hr before collection. The results are summarized in the graph. Figure S5. Treatment of different siRNAs targeted for USP5 show a similar phenotype. A: Survival assay. HeLa cells were plated at 1×105 cells per well of 6 well-plates and cultured overnight. Cells were transfected with negative control siRNA (siNC) or 2 nM siRNA for USP5 (siUSP5-02, -03 or -04) by using DharmaFECT (Thermo). After 2 days these cells were subjected to the survival assay. Cells were plated at 200 cells per 60-mm Petri dish and treated with Bleocin or methyl methanesulfonate (MMS). Cells were cultured after treatment for 11 days. Colonies were fixed and stained with 0.3% crystal violet in methanol, and the number of colonies was counted. The averages and SEDs were obtained from three dishes at each point. The error bars indicate ± SED. The P-value was calculated using Student's t-test. Results of western blot analysis after siRNA treatment are shown at the top. B: HR assay. DR-GFP cells were plated at 1×105 cells per well of 6 well-plates and cultured overnight. Cells were transfected with negative control siRNA (siNC) or 2 nM siRNA for USP5 (siUSP5-02, -03 or -04) by using DharmaFECT. After overnight incubation, these cells were transfected with pCMV-NLS-I-SceI by Lipofectamin 2000 (Life Technologies). After 2 days these cells subjected to FACS analysis (BD Accuri C6; BD Biosciences). The averages and SEDs were obtained from three independent experiments. The error bars indicate ± SED. The P-value was calculated using Student's t-test. Results of western blot analysis after siRNA treatment are shown at the top.
(DOC)
