# Supplementary material for: Ubiquitin-Specific Protease 5 Is Required for the Efficient Repair of DNA Double-Strand Breaks
Source: PLoS One. 2014 Jan 14;9(1):e84899. doi: 10.1371/journal.pone.0084899 (PMC3891734; doi:10.1371/journal.pone.0084899)
Supplement: File S1 — Figures S1–S5. Figure S1. U2OS I-SceI system for inducing DSB in a single cell; Enzymatic production of DSB at a restricted area of the nucleus. An I-SceI site was inserted next to 96 repeats of TRE. The length of an I-SceI site is 18 base pairs and there is no I-SceI site in the human genome. We introduced this construct into a U2OS cell and selected a clone which has more than 200 copies of the construct in its genome at only one position. To show the position where this construct is inserted, Cherry-TA (Cherry is a red fluorescent protein) and TA (a trans-activator which binds to TRE) were used. When we express Cherry-TA, the spot which shows the position of the construct is detected as a red focus. With expression of I-SceI, a γH2AX focus colocalized with a Cherry focus is detected. With expression of I-SceI and a GFP-tagged repair protein, Ku80, a GFP-Ku80 focus colocalized with a Cherry focus is detected. Figure S2. Treatment with an ATM inhibitor affects the foci formation of γH2AX after bleocin treatment. A: Cells were treated with 5 µg/µl of bleocin for 1 hr with or without the ATM inhibitor, KU-55933, and then cells were washed twice and fresh medium was added with or without KU-55933. After incubation for 2 hr, cells were fixed and stained with anti-phospho histone H2AX. Representative data are shown. B: % of γH2AX foci positive cells was summarized in the graph. The number of cell nuclei which contain more than five γH2AX foci was counted as foci positive cells. In each condition, more than 300 cells were counted. Figure S3. Treatment with the PARP inhibitor, Olaparib, does not affect the damage response of USP5 after laser irradiation. Percent of USP5-EGFP foci positive cells at 5 min after irradiation was summarized in the graph. The result was obtained in two independent experiments and more than 50 cells were irradiated and analyzed. Figure S4. siUSP5 treatment does not affect cell cycle progression. Cells were treated with or without siUSP5. After 3 [file pone.0084899.s001.doc]

**Supplemental Data**

**
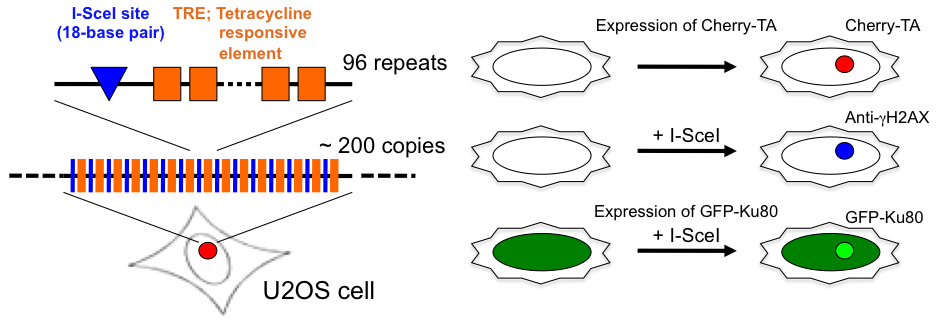
**

**Figure S1. U2OS I-SceI system for inducing DSB in a single cell; Enzymatic production of DSB at a restricted area of the nucleus.** An I-SceI site was inserted next to 96 repeats of TRE. The length of an I-SceI site is 18 base pairs and there is no I-SceI site in the human genome. We introduced this construct into a U2OS cell and selected a clone which has more than 200 copies of the construct in its genome at only one position. To show the position where this construct is inserted, Cherry-TA (Cherry is a red fluorescent protein) and TA (a trans-activator which binds to TRE) were used. When we express Cherry-TA, the spot which shows the position of the construct is detected as a red focus. With expression of I-SceI, a γH2AX focus colocalized with a Cherry focus is detected. With expression of I-SceI and a GFP-tagged repair protein, Ku80, a GFP-Ku80 focus colocalized with a Cherry focus is detected.


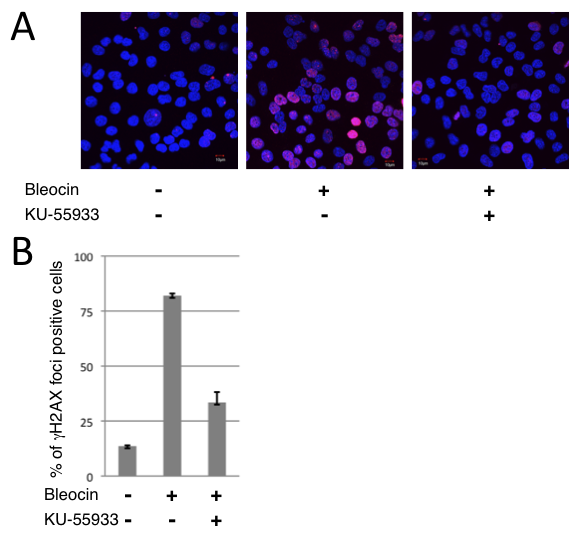


**Figure S2. Treatment with an ATM inhibitor affects the foci formation of** γ**H2AX after bleocin treatment.** **A**: Cells were treated with 5 µg/µl of bleocin for 1 hr with or without the ATM inhibitor, KU-55933, and then cells were washed twice and fresh medium was added with or without KU-55933. After incubation for 2 hr, cells were fixed and stained with anti-phospho histone H2AX. Representative data are shown. **B**: % of γH2AX foci positive cells was summarized in the graph. The number of cell nuclei which contain more than five γH2AX foci was counted as foci positive cells. In each condition, more than 300 cells were counted.


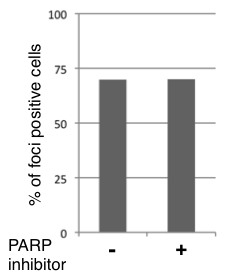


**Figure S3. Treatment with the PARP inhibitor, Olaparib, does not affect the damage response of USP5 after laser irradiation.** Percent of USP5-EGFP foci positive cells at 5 min after irradiation was summarized in the graph. The result was obtained in two independent experiments and more than 50 cells were irradiated and analyzed.


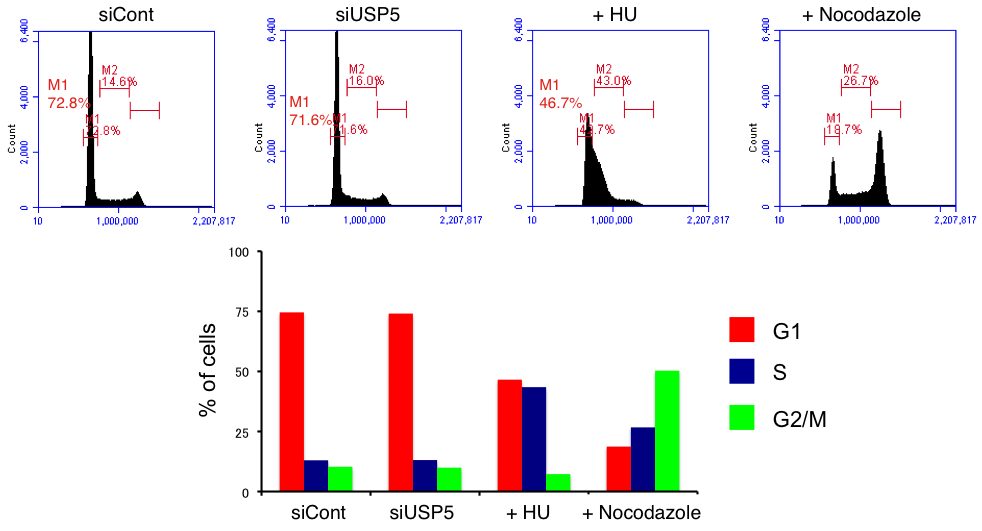


**Figure S4. siUSP5 treatment does not affect cell cycle progression.** Cells were treated with or without siUSP5. After 3 days, cells were fixed with 70% ethanol and stained by PI (Propidium iodide) and then analyzed with a C6 Flow Cytometer (Accuri Cytometers, Inc.). For a positive control, cells were treated with HU (1 mM) or nocodazole (100 µg/ml) for 24 hr before collection. The results are summarized in the graph.


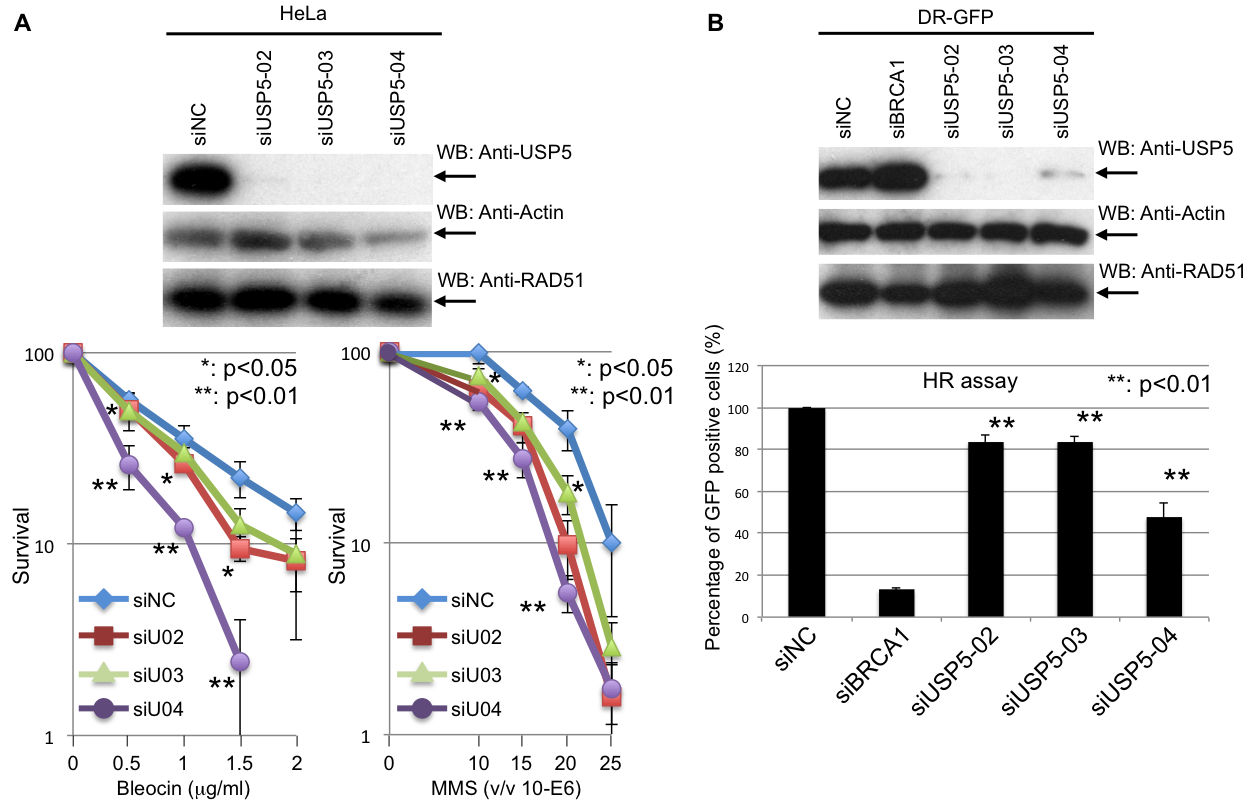


**Figure S5. Treatment of different siRNAs targeted for USP5 show a similar phenotype.** **A**: Survival assay. HeLa cells were plated at 1 x 10^5^ cells per well of 6 well-plates and cultured overnight. Cells were transfected with negative control siRNA (siNC) or 2 nM siRNA for USP5 (siUSP5-02, -03 or -04) by using DharmaFECT (Thermo). After 2 days these cells were subjected to the survival assay. Cells were plated at 200 cells per 60-mm Petri dish and treated with Bleocin or methyl methanesulfonate (MMS). Cells were cultured after treatment for 11 days. Colonies were fixed and stained with 0.3% crystal violet in methanol, and the number of colonies was counted. The averages and SEDs were obtained from three dishes at each point. The error bars indicate ± SED. The *P*-value was calculated using Student’s *t*-test. Results of western blot analysis after siRNA treatment are shown at the top. **B**: HR assay. DR-GFP cells were plated at 1 x 10^5^ cells per well of 6 well-plates and cultured overnight. Cells were transfected with negative control siRNA (siNC) or 2 nM siRNA for USP5 (siUSP5-02, -03 or -04) by using DharmaFECT. After overnight incubation, these cells were transfected with pCMV-NLS-I-SceI by Lipofectamin 2000 (Life Technologies). After 2 days these cells subjected to FACS analysis (BD Accuri C6; BD Biosciences). The averages and SEDs were obtained from three independent experiments. The error bars indicate ± SED. The *P*-value was calculated using Student’s *t*-test. Results of western blot analysis after siRNA treatment are shown at the top.
